# A complete annotation of the chromosomes of the cellulase producer *Trichoderma reesei* provides insights in gene clusters, their expression and reveals genes required for fitness

**DOI:** 10.1186/s13068-016-0488-z

**Published:** 2016-03-29

**Authors:** Irina S. Druzhinina, Alexey G. Kopchinskiy, Eva M. Kubicek, Christian P. Kubicek

**Affiliations:** Research Area Biotechnology and Microbiology, Institute of Chemical Engineering, TU Wien, 1060 Vienna, Austria; Steinschötelgasse 7, 1100 Vienna, Austria

**Keywords:** *T. reesei*, Chromosomes, Gene clusters, Telomeres, Cellulases, Proteases, Gene silencing, Siderophores

## Abstract

**Background:**

Investigations on a few eukaryotic model organisms showed that many genes are non-randomly distributed on chromosomes. In addition, chromosome ends frequently possess genes that are important for the fitness of the organisms. *Trichoderma reesei* is an industrial producer of enzymes for food, feed and biorefinery production. Its seven chromosomes have recently been assembled, thus making an investigation of its chromosome architecture possible.

**Results:**

We manually annotated and mapped 9194 ORFs on their respective chromosomes and investigated the clustering of the major gene categories and of genes encoding carbohydrate-active enzymes (CAZymes), and the relationship between clustering and expression. Genes responsible for RNA processing and modification, amino acid metabolism, transcription, translation and ribosomal structure and biogenesis indeed showed loose clustering, but this had no impact on their expression. A third of the genes encoding CAZymes also occurred in loose clusters that also contained a high number of genes encoding small secreted cysteine-rich proteins. Five CAZyme clusters were located less than 50 kb apart from the chromosome ends. These genes exhibited the lowest basal (but not induced) expression level, which correlated with an enrichment of H3K9 methylation in the terminal 50 kb areas indicating gene silencing. No differences were found in the expression of CAZyme genes present in other parts of the chromosomes. The putative subtelomeric areas were also enriched in genes encoding secreted proteases, amino acid permeases, enzyme clusters for polyketide synthases (PKS)–non-ribosomal peptide synthase (NRPS) fusion proteins (PKS–NRPS) and proteins involved in iron scavenging. They were strongly upregulated during conidiation and interaction with other fungi.

**Conclusions:**

Our findings suggest that gene clustering on the *T. reesei* chromosomes occurs but generally has no impact on their expression. CAZyme genes, located in subtelomers, however, exhibited a much lower basal expression level. The gene inventory of the subtelomers suggests a major role of competition for nitrogen and iron supported by antibiosis for the fitness of *T. reesei*. The availability of fully annotated chromosomes will facilitate the use of genetic crossings in identifying still unknown genes responsible for specific traits of *T. reesei*.

**Electronic supplementary material:**

The online version of this article (doi:10.1186/s13068-016-0488-z) contains supplementary material, which is available to authorized users.

## Background

It is well known that the organization of genes within eukaryotic genomes is non-random [1–3]. The functionally linked genes may occur in either loose groups (i.e. they are not necessarily located in immediate vicinity but enriched in several areas), or tightly packed clusters such as those involved in secondary metabolite synthesis in filamentous fungi [4] or the small secreted effector proteins in plant pathogenic fungi [5]. Regions that contain the most actively expressed genes have a higher gene density [6, 7] and a high G/C content [7]. Numerous genome-wide gene expression analyses revealed that a large portion of co-expressed genes are also co-localized in specific areas of chromosomes [8–11].

Particularly interesting are gene clusters located near telomeres—specialized sequences that terminate linear eukaryotic chromosomes by tandem arrays of simple nucleotide repeats [12]. The gene clusters found in subtelomeric regions often play roles in adaptation to an ecological niche: in yeast *Saccharomyces cerevisiae* (Saccharomycetales, Ascomycota), they contain families of genes involved in sugar utilization [13], and in the protozoa *Plasmodium falciparum* (Chromalveolata, Apicomplexa) and *Trypanosoma brucei* (Excavata, Euglenozoa) or the yeast-like fungus *Pneumocystis jiroveci* (former *P. carinii,* Pneumocystidales, Ascomycota), all parasites of humans, subtelomeric regions contain families of variant and frequently paralogous genes encoding surface proteins [15–17]. This allows to switch the expression among different gene copies which enables to escape the hosts’ immune system [18, 19].

*Trichoderma reesei* (Hypocreales, Ascomycota) is a major source for the industrial production of plant cell wall degrading enzymes that are applied in the pulp and paper, food and textile industries, as well as for the conversion of plant biomass materials into biorefinery products [20]. Although the production of such enzymes by *T. reesei* has been considerably improved through genetic modification of industrial strains [21], the availability of ecological genomic approaches (i.e. the integration of –*omic* data with knowledge on the ecology of the organism) would open a gateway for further biotechnological developments. Despite the fact that *T. reesei* is the best-studied species in the genus and thus was the first whose genome has been sequenced [22], our understanding of its role in the ecosystem and nutritional preferences remains controversial. The known natural habitat for the about 20 wild-type strains is dead wood [23], which suggest saprotrophy what would be in line with a superior production of extracellular enzymes for degradation of plant biomass. However, in vitro studies indicate that this fungus is also capable to parasitize other fungi [23], and thus it maintained the innate trait of the genus *Trichoderma,* mycotrophy including mycoparasitism [24].

Interestingly, the plant cell wall degrading and other carbohydrate-active enzymes (CAZymes) of *T. reesei* occur in loose groups [22]. Some of these groups are also co-regulated [25, 26], and their co-expression has successfully been used to identify further genes encoding solute transporters or transcription factors that are involved in the induction and expression of CAZymes [25, 27]. However, since a chromosomal map of *T. reesei* was not available, all above studies had been done with the 98 scaffolds of the *T. reesei* v2.0 genome database (http://genome.jgi-psf.org/Trire2/Trire2.home.html; Department of Energy, Joint Genome Institute, USA). It is therefore possible that the full picture of these loose groups has not yet been obtained.

*Trichoderma reesei* contains seven chromosomes with sizes from 2.8 to 6.9 Mb [28, 29]. Recently, Marie-Nelly et al. [30] using genome-wide chromosome conformation capture (3C) data, assembled the 98 scaffolds to seven chromosomes. Their data showed that some of the original scaffolds were misassembled and fragments were in fact parts of different chromosomes. They also noted that some of the CAZyme clusters were apparently located close to the chromosomal ends (subtelomeric regions), but did not further investigate this finding [30].

So far, the gene arrangements and expression dynamics of chromosomes from non-clinical filamentous fungi have not been investigated. In addition, only little attention has been paid to these two points with respect to subtelomeric regions [31–34]. The hypothesis of this work was that a detailed analysis of the clustering and expression dynamics of genes on the *T. reesei* chromosomes may provide us with new insights into the gene inventory important for habitat specialization of this fungus. In addition, the availability of fully annotated chromosomes may be a valuable tool to identify the genes for specific traits by genetic crossings.

We will here present a genome-wide view of the organization of the chromosomes of the cellulase producer and mycoparasitic fungus *T. reesei.*

## Results

### General properties of the *T. reesei* chromosomes

We have used the GRAAL-supported assembly of the *T. reesei* scaffolds into seven chromosomes [30] to map the genes of *T. reesei* v2.0. To this end, we adopted the chromosome nomenclature of *Neurospora crassa* (Sordariales, Ascomycota) [35] and thus categorized them with Arabic numbers, starting with the largest chromosome. The sizes, number of genes and gene densities of these seven chromosomes is given in Table 1: because of a duplication of an area in chromosome seven containing the rRNA gene clusters and ten additional genes [30] the *T. reesei* genome finally contains 9194 ORFs.

**Table 1:**
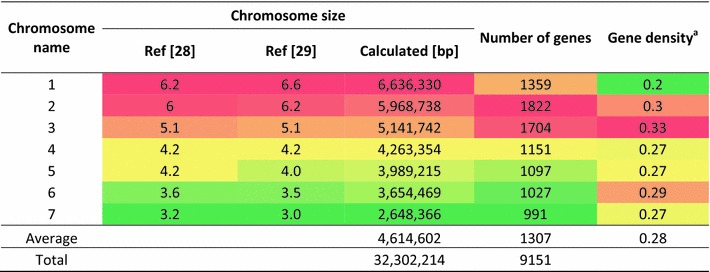
The seven chromosomes of *T. reesei*

^a^Genes per 1 kb

As reported previously [30], scaffolds 57, 63, 70, 72 and 87—which make up for a total of 58 kb (0.17 % of the total genome)—were missing in the chromosome assembly. Only two contained ORFs (scaffold 63: Trire2:71126 http://genome.jgi.doe.gov/cgi-bin/dispGeneModel?db=Trire2&id=71126 and Trire2:112687 http://genome.jgi.doe.gov/cgi-bin/dispGeneModel?db=Trire2&id=112687; scaffold 70: Trire2:71173 http://genome.jgi.doe.gov/cgi-bin/dispGeneModel?db=Trire2&id=71173) which all encoded orphan genes of unknown function. We found no transcripts for these three genes during growth of *T. reesei* QM 9414 on glucose, glycerol, lactose and cellulose in submerged culture, upon cellulase induction by sophorose, during growth on plates with glucose as a carbon source, or during conidiation (C. P. Kubicek, unpublished data). The biological role and genomic location of these ORFs is thus unclear.

The size of the chromosomes, determined in silico, correlates mostly well with their sizes derived from CHEF analysis ([28, 29]; Table 1) when an experimental error of 5 % is assumed. Only the size of Chr7 exceeds this frame, as the in silico assembly resulted in a 300–500 kb smaller size then predicted by CHEF. The gene density on individual chromosomes was also different, ranging from 0.21 to 0.33 gene per 1 kb (Table 1).

Eukaryotic chromosomes are linear molecules which contain telomeres at their terminal ends that serve to protect against loss of DNA from lagging strands during replication. In order to confirm the completeness of the in silico assembled *T. reesei* chromosomes, we searched for the presence of telomere repeat sequences ([TTAGGG]_*N*_) at the ends of seven chromosomes. We found telomere repeats on two of them (Chr1 and Chr5) on both termini, one repeat on the 5′ end of Chr3 and one repeat on the 3′ end of Chr4. They were identical to six of the seven telomere repeats previously reported in the *T. reesei* genome [22]. The seventh one resides on scaffold 87 which could not be aligned during the GRAAL assembly (vide supra). No telomere repeats could be identified at either ends of Chr2, Chr6 and Chr7. The typical length of the identified telomere repeats was 84–102 nucleotides (14≤ *N* ≤ 17), with the exception of Chr3 (*N* = 5).

The chromosome area next to the telomeric ends typically contains AT-rich sequences of 1000–3000 nucleotides. In order to find out whether they are present at the putative ends of the seven *T. reesei* chromosomes, we used *Z*-curve analysis, a window-less approach [36], to plot the GC content over the terminal 50 kb’s (Additional file 1). We identified up to 2.8 kb long nucleotide stretches with a GC percentage of 10–18 % at both termini of four chromosomes, and on one terminus each of three others. They were absent, however, from the 5′ end of Chr3, and the 3′ end of Chr6 and Chr7. As a control, we randomly sampled one hundred 20 kb regions from non-terminal areas of the seven chromosomes. Only 9 % displayed an AT content >80 %, whereas the average DNA had a GC content of 51.5 [±8]  %.

In the subtelomeric regions of chromosomes from *Magnaporthe oryzae* (Magnaporthales, Ascomycota) [32, 33] and *Aspergillus nidulans* (Eurotiales, Ascomycota) [31], certain sequences are found at several locations of chromosome ends. In agreement with finding in *N. crassa* [34], however, the terminal 20 kb’s on each side of the seven chromosomes of *T. reesei* did not display any regions of similarity to one another. Also consistent with *N. crassa* but in contrast to other fungi, we failed to detect telomere-linked helicase genes that have reported to be present in the subtelomeric regions of several filamentous fungi [32, 33, 37, 38], and we did not detect any telomere-associated, short tandem repeats. Thus, like *N. crassa* but unlike several other fungi, *T. reesei* does not have a typical subtelomeric region that is defined by specific sequences.

### Clustering of functionally related genes over the chromosomes of *T. reesei*

**Fig. 1:**
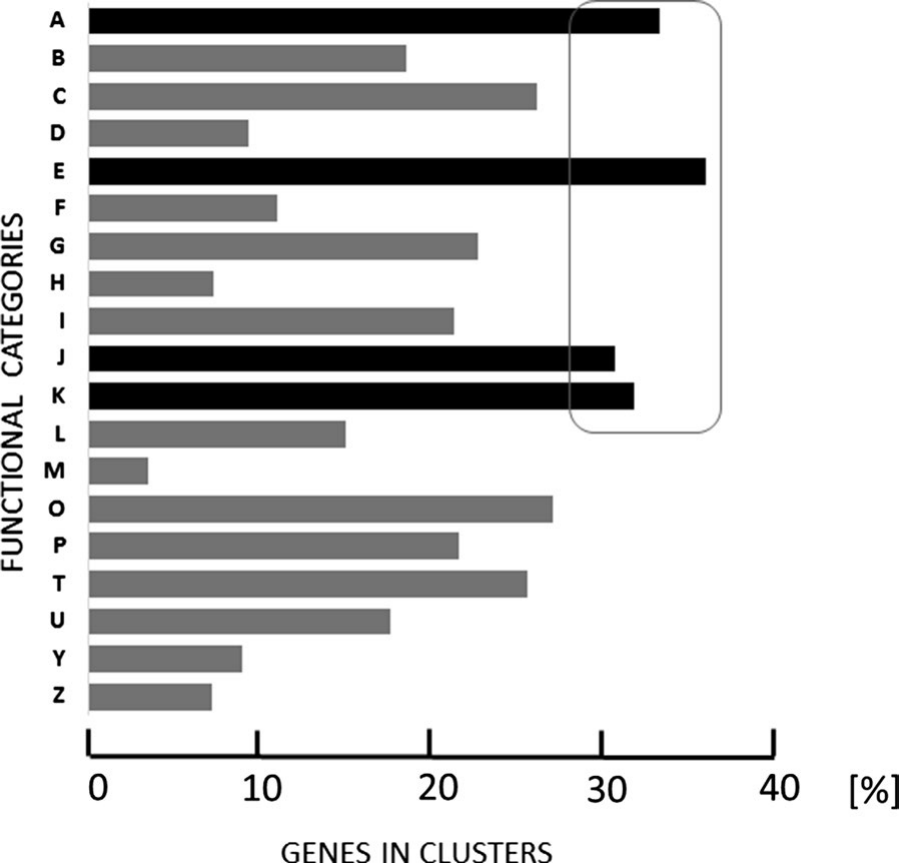
Percentage of genes clustered according to the cellular function, abbreviated as used in the KOG categorization: *A* RNA processing and modification (346); *B* chromatin structure and dynamics (136); *C* energy production and conservation (316); *D* cell cycle division and control (196); *E* amino acid transport and metabolism (290); *F* nucleotide transport and metabolism (95); *G* carbohydrate transport and metabolism (346); *H* coenzyme transport and metabolism (95); *I* lipid transport and metabolism (297); *J* translation, ribosomal structure and biogenesis (339); *K* transcription (393); *L* replication, recombination and repair (192); *M* Cell wall/membrane/envelope biogenesis (87); *O* Posttranslational modification, protein turnover, chaperones (559); *P* inorganic ion transport and metabolism (177); *T* signal transduction mechanisms (516); *U* intracellular trafficking, secretion, and vesicular transport (313); *Y* nuclear structure (106); *Z* cytoskeleton (180). The numbers in *brackets* indicate the total number of *T. reesei* genes in the given category. The *black boxes* indicate those categories that contain more than 30 % of genes in clusters

In view of the accumulating evidence that many functionally related genes are clustered in eukaryotic genomes (see “Background”), we used the assembled chromosomes to test whether this is also the case in *T. reesei.* To this end, we grouped genes according to 19 KOG categories (comprising 5754 genes), and mapped them on the seven chromosomes. We then divided the total number of ORFs in the *T. reesei* genome (9194) by the number of genes falling into one of the 19 KOG categories. This value was then taken as the theoretical average distribution of genes of this functional category (e.g. if the value was 50, we should theoretically find a gene once within fifty subsequently ordered genes). We then defined a cluster as the subsequent occurrence of at least three genes that were on the average separated from each other by less than a fifth of the above determined average distribution number (e.g. in the above example at least three genes within a stretch of thirty genes). In KOG families A, B, C, E, G, I, J, K, O, P and T, more than 20 % of the genes were found in such clusters (*p* < 0.05). Four (A: RNA processing and modification; E: amino acid transport and metabolism; J: translation and ribosomal structure and biogenesis and K: transcription) contained significantly (*p* = 0.002) more of their genes in genomic clusters than the other fifteen categories (Fig. 1).

**Fig. 2:**
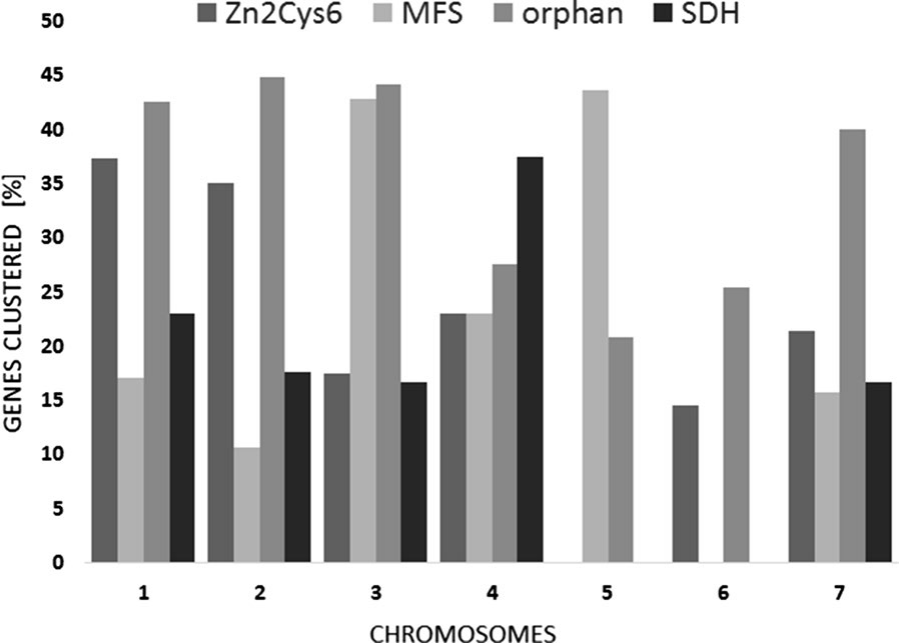
Clusters of selected gene groups that are overrepresented in *T. reesei*, on the seven chromosomes

We then extended this analysis to those gene families that are expanded in the *Trichoderma* genomes [39]. As shown in Fig. 2, the clusters encoding Zn2Cys6 transcription factors, and the major facilitator superfamily (MFS) transporters indeed exhibited a significant (*p* = 0.0033) degree of non-random distribution. These clusters were unevenly distributed over the chromosomes and on some exceeded even 40 % of the genes of the respective family. The short-chain dehydrogenase/reductases also showed some, albeit lower, cluster proportion (15.9 %). All of the other expanded gene families (see [39] for the full list), however, showed random distribution (data not shown).

### A third of the CAZyme genes is non-randomly distributed in the *T. reesei* chromosomes

*Trichoderma reesei* CAZymes—including cellulases, hemicellulases and chitinases—have been previously reported to be located in clusters (= regions “containing a statistically higher proportion of a particular gene family and must begin and end with a gene from the family in question” [22]). In order to assess the location and structure of these clusters on the chromosomes, we used the following rationale (ignoring the finding of scaffold-based clusters): *T. reesei* contains 228 CAZymes [26], and—under the assumption of a completely random distribution within the 33.3 Mb of the genome—each CAZyme encoding gene should on the average be found once per 146.052 kb (corresponding to one CAZyme per every 41 ORFs, if the mean value of one gene per 3.56 kb is used for calculation; see Table 1). Therefore, in order to identify the non-randomly distributed CAZymes, we used a sliding window approach to screen the chromosomes for the presence of at least three CAZyme encoding genes that are separated by eight or less other ORFs. Eight was chosen because it represents a fivefold excess over the average distribution of one CAZymes per 41 ORFs and provides a significance of *p* = 0.001. Twenty such clusters, comprising 71 of the 228 CAZyme encoding genes (and swollenin, which has cellulose hydrolase activity but is not included in the CAZyme classification [40]) were found (average *p* for all 20 clusters 5.3e−5; Additional file 2). Eighteen further genes were present as pairs separated by ≤3 other genes. The remaining 139 CAZyme encoding genes were randomly distributed throughout the chromosomes. Five were located close to the chromosomal ends, but not clustered with the others.

An analysis of the location of the 20 clusters on the chromosomes revealed an interesting fact: five clusters (comprising 17 genes; present on Chr3, Chr4, Chr6 and Chr7) were located within 50 kb from the chromosome end. For convenience, we will further call these clusters CEC (chromosome end clusters). Further seven clusters (each one on Chr1, Chr4, Chr5, Chr6, Chr7 and two on Chr2; comprising 26 genes) were present between 65 and 150 Kb’s from the chromosome ends. We will further call these clusters NCEC (near chromosome ends clusters). This indicates that approximately half of the CAZyme clusters are located within a distance of 1–4 % of the total chromosome length from the respective chromosome ends. The remaining eight clusters were located within the central part of the chromosomes (MCC, middle-located chromosome clusters).

### Genes encoding small secreted cysteine-rich proteins cluster together with CAZyme genes

The genomes of biotrophic (for instance, plant pathogenic and nematode trapping) fungi harbour clusters of small secreted cysteine-rich proteins (SSCPs) [5, 41, 42]. The *Trichoderma* genomes also contain a significant number of such proteins [43]. We therefore wondered whether the CAZyme clusters in the *T. reesei* genome may actually be part of clusters of secreted, particularly SSCP proteins. To investigate this we applied the stringent criteria of Kämper et al. [5] and screened for groups of at least three adjacent genes encoding secreted proteins or groups containing more than three genes with at most one gene encoding a non-secreted protein in between. Indeed, 42 such gene clusters containing genes for 148 secreted proteins were identified, which corresponds to 16.9 % of all secreted proteins in this fungus [43]. This value is lower than in the nematode trapping fungus *Monacrosporium haptotylum* (Helotiales, Ascomycota) (27.2 %; [41]), but in the same range as in *Ustilago maydis* (Ustilaginales, Basidiomycota) (18.6 %; [5]). We must note, however, that the gene clusters in these both fungi range up to 11 and 26 genes, whereas the gene clusters of *T. reesei* consist of 3–6 genes only. The gene composition of the 42 *T. reesei* SSCP clusters is given in Additional file 3: CAZymes and unknown proteins accounted for the highest number of genes (34 and 35, respectively), and it was of interest to see that all the six CAZyme clusters present in the *T. reesei* CEC regions were parts of the SSCP clusters. SSCPs, proteolytic enzymes and orphan proteins constituted 15, 13 and 13 genes, respectively (Additional file 3). Thereby the clusters containing SSCPs also contained a third of all CAZymes present in the 42 clusters, whereas the CAZyme and protease containing clusters exhibited no significant (*p* > 0.05) bias towards any of the other gene groups (Table 2).

**Table 2:** Gene co-occurrence of the *T. reesei* SSCPs clusters

	Clusters	Cluster members				
		SSCP	CAZyme	Protease	Unknown	Orphan
SSCP	11	*15*	11	1	4	4
CAZyme	23	5	*34*	7	14	5
Protease	12	1	9	*14*	5	3

The numbers show how many of the other genes were co-localized in gene clusters containing the gene shown in the most left column

*SSCP* small secreted cysteine-rich proteins

### Biased gene distribution in CEC and NCEC

For several fungi, the regions adjacent to the chromosome ends have been reported to contain genes relevant for adaptation to the ecological niche [13, 15–19]. Our inventory of gene families that are present in *T. reesei* CEC and NCECs indeed revealed a pattern of overrepresentation of several gene families encoding polyketide synthases (PKS), PKS–non-ribosomal peptide synthase (NRPS) fusion proteins (PKS–NRPS), secreted proteases (*p* = 0.021) and amino acid permeases (*p* = 0.009). In addition, CEC was enriched in genes encoding unknown short chain dehydrogenases and reductases, PTH11 receptors, cytochrome P450 monooxygenases and proteins related to iron scavenging (Table 3). The latter included two siderophore transporters, one ferric reductase and a siderophore biosynthesis cluster which includes a siderophore synthase and five enzymes of the siderophore biosynthetic pathway (see below). With the exception of the iron scavenging proteins, which because of the presence of several ferric reductases, iron transporters and siderophore transporters make up for a total of 25 gene, however, all failed statistic tests of significance (*p* = 0.015 vs. *p* > 0.05).

**Table 3:**
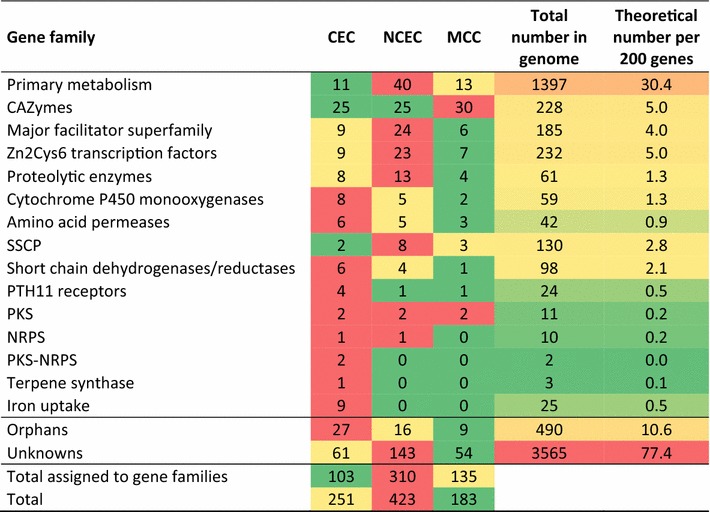
Distribution of *T. reesei* gene groups in the three clusters CEC, NCEC and MCC

Martinez et al. [22] already noted the occurrence of PKS and NRPS encoding genes within the loose CAZyme clusters. Five of the 11 PKS genes [44], 2 of the 10 NRPS genes, both genes encoding PKS–NRPS hybrids and one of the three terpene synthases were located within the two CEC and NCEC clusters (Table 4). Yet only one PKS, the pigment forming PKS4 ([45], Trire2:82208), both PKS–NRPS (Trire2:58285 and Trire2:59315), one NRPS (Trire2:71005) and one terpene synthase (Trire2:112028) were located within the CECs.

**Table 4:** Occurrence of genes encoding secondary metabolite synthases in loose CAZyme clusters in *T. reesei*

	CEC	NCEC	MCC	Chr	Annotation
PKS	82,208			4	PKS4
		65,172		7	PKS1
		105,804		1	PKS3
			73,621	5	PKS singlet 11
			73,618	5	PKS singlet 10
NRPS	71,005			5	SID4
		60,458		2	Ortholog of SirP
PKS–NRPS	58,285			2	
	59,315			1	
Terpene synthase	112,028			3	

Numbers specify the corresponding Trire2 protein IDs. Annotations of PKSs are taken from Baker et al. [44]. Location (Chr) specifies the chromosome on which the gene is located

The putative products formed by the two PKS–NRPS were determined by antiSMASH [http://antismash.secondarymetabolites.org/] [46] and are shown in (Additional file 4). Trire2:59315 and Trire2:58285 have an essentially similar domain structure and share 55 % functional conservation of amino acids. However, their neighbouring genes are essentially different (Additional file 4), whereas Trire2:59315 also contains a single PKS (Trire2:105804), a single NRPS and several putative processing enzymes, Trire2:58285 clusters with genes encoding a P450 monooxygenase, a dienoate reductase, an alcohol dehydrogenase, an *N*-acetyltransferase and a Zn2Cys6 transcription factor.

**Fig. 3:**
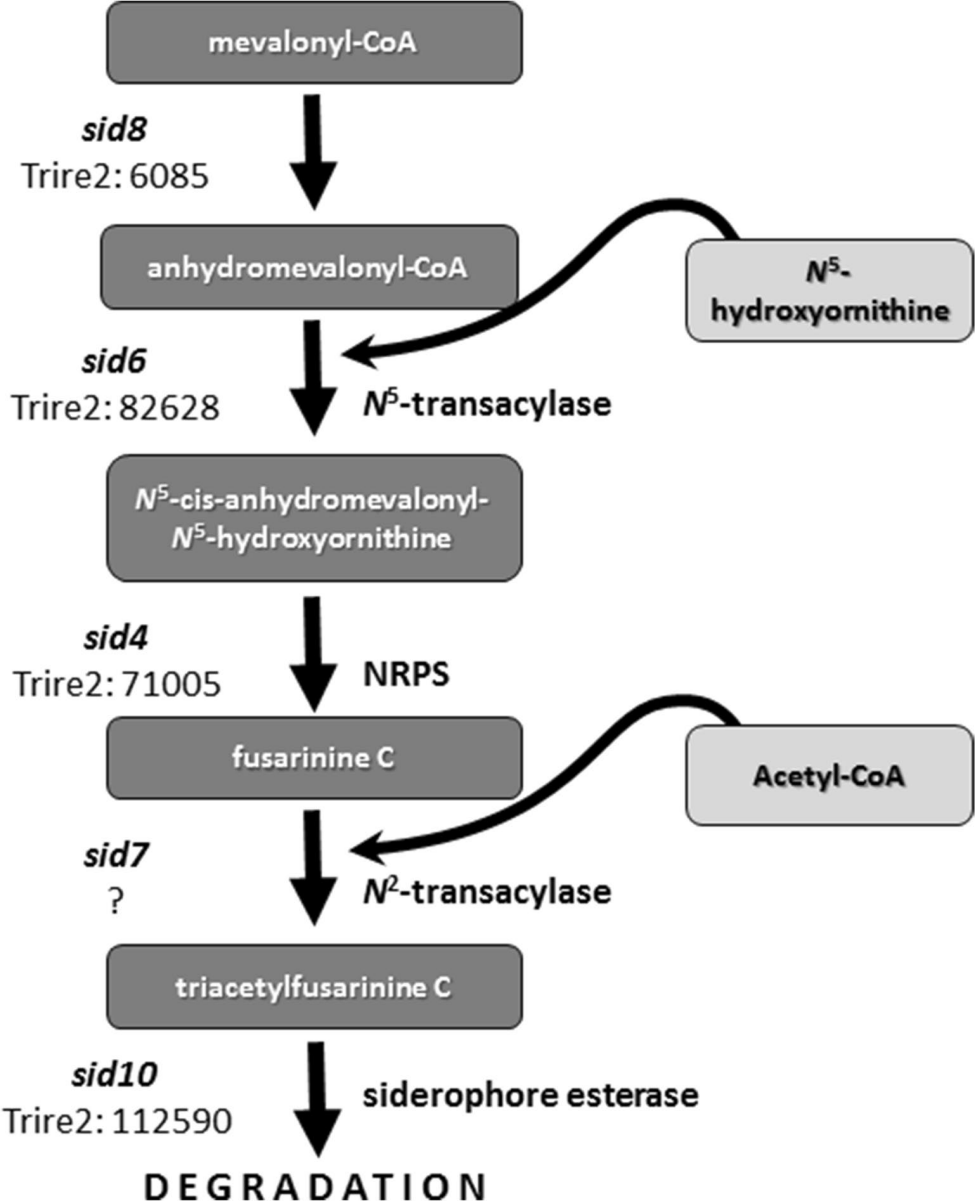
Involvement of the genes found on the 3′ end of Chr5 in *T. reesei* in the biosynthesis of the siderophore fusarinine. Pathway structure and gene nomenclature was taken from *Aspergillus* *fumigatus* [47]. The gene nomenclature for *T. reesei* was adapted using the corresponding numbers instead of letters, in order to follow the *Neurospora* gene nomenclature [35]. The “?” indicates that a *sid7* orthologue could not yet be found, as blastp of *T. reesei* with the respective *A. fumigatus* SidG protein resulted in no hits (data not shown)

The only NRPS present in the CEC mentioned above is the siderophore synthase *sid4* (Trire2:71005, Chr5), which is situated in a gene cluster consisting of two enzymes of the biosynthesis of the SID4 substrates (the transacylase SID6 and the mevalonyl-CoA-dehydratase SID8), a siderophore transporter, an ABC transporter and the siderophore esterase SID10 (Fig. 3). The gene encoding the l-ornithine *N*5-oxygenase, which starts this pathway, is missing from this cluster but is present immediately beneath the orthologue of the enzyme forming the second *T. reesei* siderophore, the ferricrocin synthase SID3 (Trire2:69946), 200 kb from the 5′ end of Chr3 (data not shown).

The NRPS present in NCEC (Trire2:60458, Chr2) is an orthologue of SirP which synthesizes the epipolythiodioxopiperazine phytotoxin sirodesmin PL in *Leptosphaeria maculans* (Pleosporales, Ascomycota) [48].

Interestingly, the cluster for high affinity nitrate assimilation, comprising *nrt2* (high affinity nitrate transporter), *nit1* (nitrate reductase) and *nir1* (nitrite reductase), which has been acquired by *T. reesei* by horizontal gene transfer from smut fungi [49], is located in CEC at the 3′ end of Chr3.

The CEC region also contained 68 (class I) retrotransposons and 87 (class II) DNA transposons. Transposable elements most frequently found were LTS-Copia and LTR-Gipsy elements in class I (16 and 20, respectively), and DNA/Mariner elements (59) in class II (Additional file 5).

### Gene clustering does not influence gene expression in *T. reesei*

**Fig. 4:**
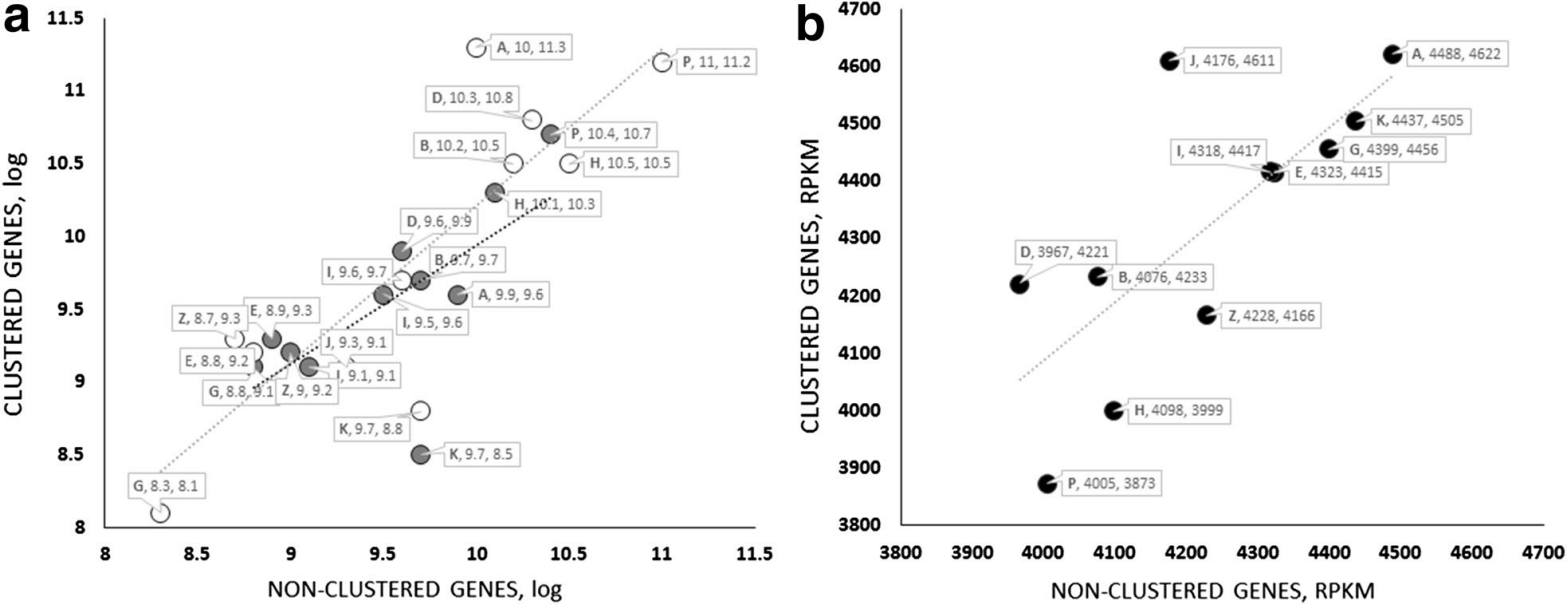
*Scatter plot* of mean expression values of clustered and non-clustered genes in different functional groups during growth on **a** d-glucose (*filled circles*) and lactose (*open circles*) and **b** glycerol. *Dotted lines* indicate linear trend lines. Mean expression values were calculated from the log hybridization intensity (for d-glucose and lactose) or RPKM (kilobase of exon per million mapped sequence reads) for glycerol. Standard deviations for the mean values were all <28 %

The clustering of genes in a genome is generally believed to enable a coordination of gene expression [10, 50]. We therefore investigated whether this would indeed be the case for *T. reesei*. To this end, we made use of available transcriptomic data for *T. reesei* grown on glucose, glycerol and lactose, and calculated the mean expression level of clustered and non-clustered gene categories shown in Fig. 1 (vide supra). However, as can be seen from Fig. 4, no significant differences were noted between clustered and non-clustered genes, as well as between different gene categories. We conclude that the genomic clustering does not result in an enhanced gene expression, at least under the conditions investigated.

### Genes located near the chromosome ends results in lower basal transcription levels

**Fig. 5:**
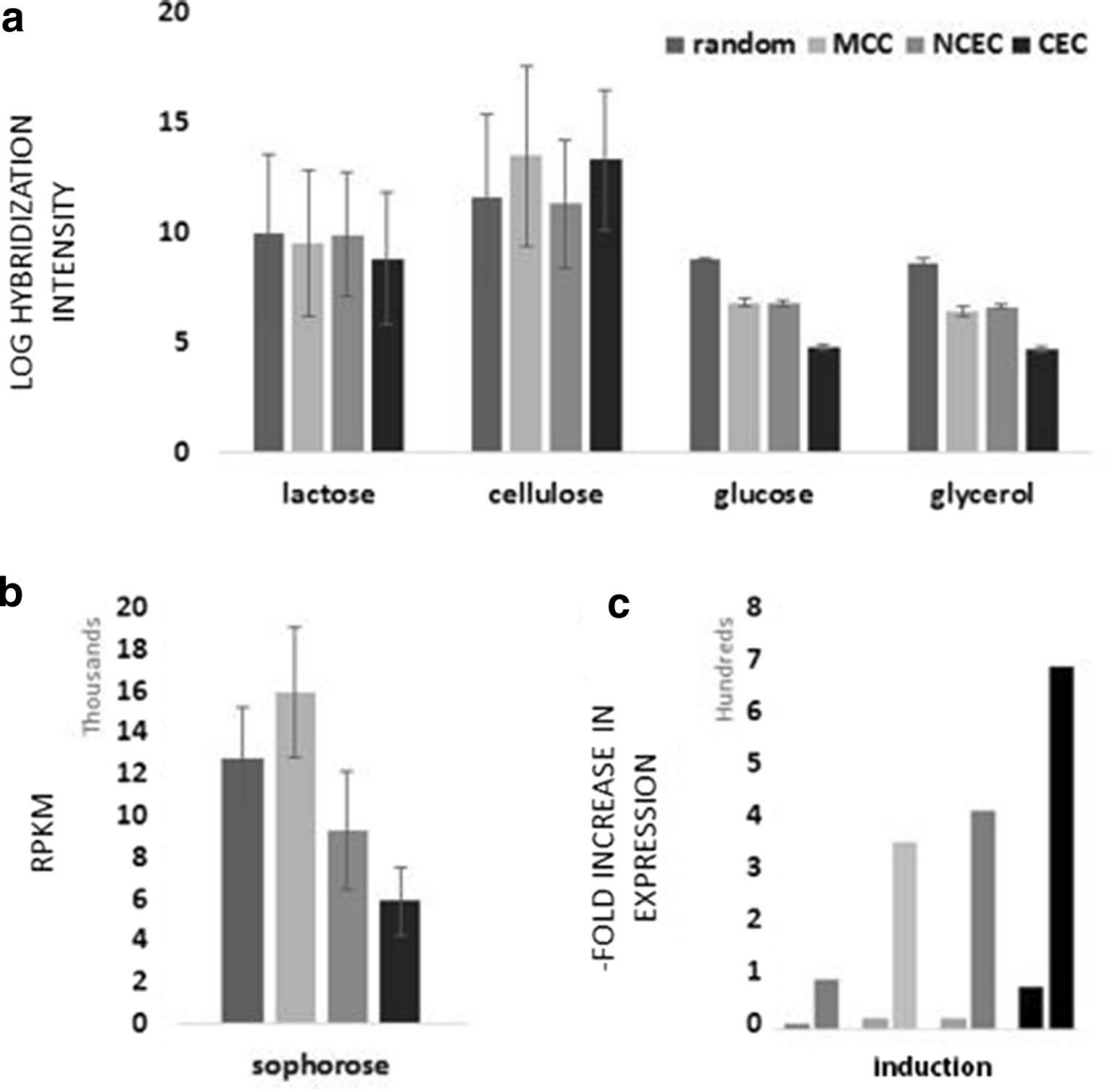
Mean expression values of CAZymes in *T. reesei* clusters during incubation on different carbon sources. **a** Mean expression value, calculated from the log hybridization intensity (for oligonucleotide arrays); **b** Mean expression value calculated from RPKM (kilobase of exon per million mapped sequence reads) of RNA deep sequencing (*asterisk*); **c** “Fold” increase of expression on cellulose (*left bar*) and lactose (*right bar*) relative to glucose. The expressions of the genes encoding *cel7A, cel6A* (both CEC) and *cel7B* (MCC) were omitted from the calculation as their expression and induction values were >100-fold higher than those of the other genes and corrupted the calculations and statistics

The occurrence of some CAZyme genes in CEC, NCEC, MCC and random distribution of the others prompted us to investigate whether this different location would be reflected in differences in gene expression. To this end, we made use of our previous transcriptome data of *T. reesei* grown on glucose and glycerol (conditions not inducing CAZymes) and on the cellulase-inducing carbon sources such as cellulose (= pretreated wheat straw), lactose and sophorose. The five non-clustered genes present within the 50 kb terminal area were included in CEC for convenience. A full description of the transcriptomes formed under these conditions has been published [51–53]. Looking for genes that were at least greater than twofold higher expressed than on glucose, we identified 67 sophorose-, 90 lactose- and 117 cellulose-induced genes. In view of the total number of CAZymes in *T. reesei*, this represents about a third to a half of the total CAZyme inventory. We then aligned these individual transcripts to the four categories CEC, NCEC, MCC and “randomly distributed genes”, and calculated the mean expression level of genes in these four groups. Figure 5a shows that there were no significant differences (*p* > 0.05) in the expression level between CEC, NCEC, MCC and the randomly distributed genes on lactose or cellulose, although the latter condition was characterized by a higher number of transcripts (*p* = 0.031). On sophorose, highest (Fig. 5b) average transcript levels were observed with genes from MCC and lowest levels for genes in CEC .

Significant differences, however, were obtained when the mean level of induction was compared between the four groups: on both lactose and cellulose, the lowest induction levels were obtained for the randomly located genes (*p* = 0.021), and the CEC genes yielded the higher value (*p* = 0.012) (Fig. 5c). The same trend was seen on sophorose with the exception that NCEC exhibited the highest degree of induction. The comparable transcript levels but significantly different degrees of induction suggested that the basal expression levels of the genes in the four clusters must be significantly different. In fact, the CEC genes displayed a basal transcription level on glucose and glycerol that was two orders of magnitude lower than that of the other clusters, and this was independent of whether microarray data (lactose, cellulose) or RNA deep sequencing data (sophorose) were used for evaluation (Fig. 5a, b). The only difference noted was that the NCEC and MCC genes displayed a similar basal expression level in the microarray data, whereas this level of MCC was as high as that of the randomly distributed genes in the RNA deep sequencing results. Irrespectively of this, the NCEC genes have a much lower basal expression level than the others.

### The *T. reesei* chromosome ends are underrepresented in epigenetic gene activation but overrepresented in gene silencing

**Fig. 6:**
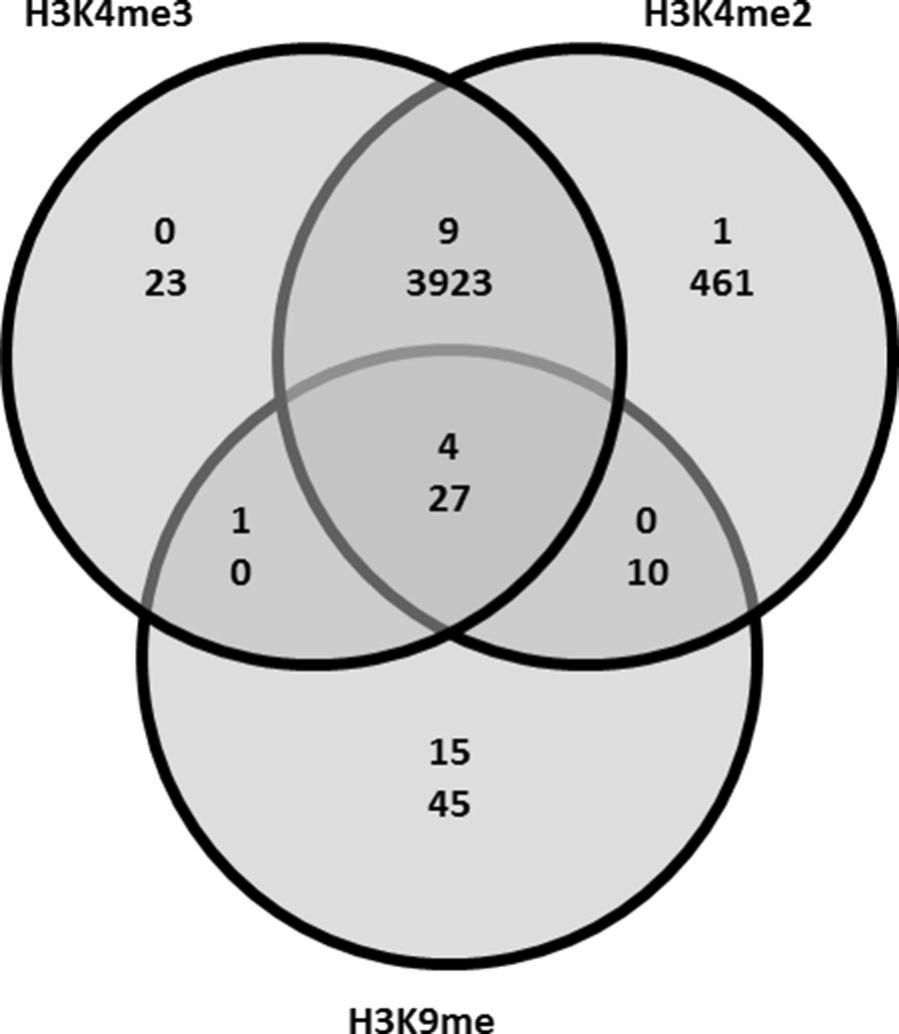
Numbers of histone H3 methylations in CEC (*large font*) and all seven chromosomes (*smaller font*) without CECs

The above data could be due to a different epigenetic state of the *T. reesei* chromosome ends. To test this, we analysed the topology of histone H3 modifications on the *T. reesei* chromosomes. We have previously investigated histone modifications by whole-genome ChIP-seq data, using antibodies against histone modifications known to be associated with transcriptionally active (H3K4me2 and -me3) or silent (H3K9me3) chromatin [54]. We now mapped these histone modifications on the chromosomes. The results showed that transcription activating H3 modifications were strongly underrepresented in CECs (Fig. 6): the total chromosome exhibited 4489 modifications, of which 3923 (corresponding to 43.8 % of all genes on the chromosomes) were due to H3K4me3 and H3K4me2. The higher number of H3K4me2 than H3K4me3 (4421 vs. 3973 genes) is in contrast to *N. crassa* [55]. Since all reads were normalized based on sequencing depth, this suggests a greater abundance of that epitope. In contrast, only 11.5 % of the genes within the subtelomeric ends showed activating histone modifications.

The opposite finding was obtained when only the H3K9 methylations were counted: 82 genes (= 0.91 %) with this type of modification were detected on the chromosome, whereas 20 of the genes at the chromosome ends (= 7.9 %) were K9 methylated. This agrees with the interpretations that the chromosome ends are subject for gene silencing by H3K9. It was thereby interesting to find that the H3K9 methylations near the chromosome ends were exclusively found in CEC, and the adjacent NCEC region was completely devoid of them (data not shown).

### Genes located at the chromosome ends show enhanced expression during conidiation and interaction with another fungus

In order to test whether the subtelomeric genes in *T. reesei* are related to the fitness (cf. [56]), we analysed the chromosomal location of genes that are significantly upregulated during the onset of interaction with *Thanatephoruscucumeris* (*Rhizoctoniasolani*, Cantharellales, Basidiomycota; [57]), and of genes that are significantly upregulated at the onset of conidiation [58]. The results, shown in Table 5, reveal that indeed, expression of genes located within CEC are greater than fivefold enriched in the presence of *T. cucumeris* over those located on MCC (*p* = 0.0014). Also, genes that were strongly upregulated at the onset of conidiation were enriched both in CEC and NCEC (*p* = 3.2e−4 and 0.001, respectively).

**Table 5:**
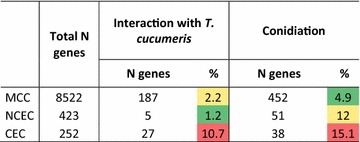
Number of clustered and non-clustered genes significantly expressed during interaction with another fungus and conidiation in *T. reesei*

Greater than twofold over the control; data taken from Refs [57] and [58]

% specifies the percentage of genes that are significantly regulated within the total gene number of the given category

Conidiation was the condition under which most of the SSCPs—120 of the 148 genes (81 %)—displayed increase gene expression (Table 6). In contrast, only 64, 33 and 9 genes were upregulated during growth on cellulose, lactose and cellulase induction by sophorose, respectively. Only five SSCP-encoding genes were expressed during confrontation with *T. cucumeris*.

**Table 6:** Expression of SSCP clusters during CAZyme induction, conidiation and fungus–fungus interaction

	148 genes clustered in 48 clusters				
	Genes	%	Clusters	%	
Cellulose	64	43.2	9	18.7	31 genes in 9 clusters,
Lactose	33	22.3	9	18.7	Lactose incomplete (16)
Sophorose	9	6	0		
Mycoparasitism	5	3.3	0		
Conidiation	120	81	26	54.2	90 genes in 26 clusters

Conditions for CAZyme induction were the same as those given in Table 7

## Discussion

In this work, we analysed the organization and transcriptional dynamics of the seven chromosomes of *T. reesei*. Although—based on the absence of diagnostic sequences—two chromosomes lack telomeres at one and three chromosomes at both ends, the correspondence of the experimentally determined size of the individual chromosomes and the size calculated from the nucleotide sequence suggests that at least the assembly of six chromosomes is nearly complete. In addition, all but Chr7 displayed an AT-rich region similar in size to that which separates the subtelomeric region from the neighbouring genes in *N. crassa* [34]. These AT-rich regions are likely the results of repeat-induced point mutation or RIP, a mechanism that detects duplicated sequences during sexual recombination and changes CG to TA base pairs, thereby causing an excess of TpA and a deficiency of CpA dinucleotides. This has been demonstrated in *N. crassa* [34], and we obtained similar results for *T. reesei* too (C.P. Kubicek, unpublished data). The genetic prerequisites for the occurrence of RIP in *T. reesei* have been demonstrated [39]. We are therefore confident that our analysis indeed fully covered at least six whole chromosomes and their gene content. The identification of the chromosome ends on Chr7 will likely require more sophisticated approaches, which were beyond the purpose of this study. The difficulty to assemble chromosome ends from sequence data, even with the aid of specific cosmid clones and RFLP analysis, has been reported in other fungi as well [34].

It is now well documented in various eukaryotes that the order of a significant gene in their genomes is not random, and particularly genes that share a similar expression pattern tend to occur at least in loose clusters [1–3]. In yeast, essential genes form large clusters that are located in regions of low recombination [59]. This co-evolution of gene order and recombination is considered to be advantageous when the fitness of an allele at one locus depends on the genotype at another locus [4]. Our data on *T. reesei* show that such a clustering also occurs in filamentous fungi: we found that most functionally related gene categories exhibited some degree of clustering, but those involved in RNA processing and modification, amino acid transport and metabolism, translation and ribosomal structure and transcription—which can be considered as essential to the cell—showed the highest degree of clustering. In addition, these clusters were not found in the chromosome ends. This is in perfect agreement with data from other organisms (vide supra). In contrast, we did not find any difference in the expression of clustered and non-clustered members of various gene categories, and in fact the differences in expression between genes of the same group, clustered or not, were significant. This agrees with other studies on eukaryotic genomes, and suggests that indeed the positioning of important or even essential genes into areas of low recombination may be the true reason for gene clustering, as already discussed in [59]. This further supports the view that the CAZymes appear to be of importance to the fitness of *T. reesei* (see below).

Because *T. reesei* is a major producer of cellulolytic and hemicellulolytic enzymes for industry, we were especially interested in the analysis of genomic clusters of CAZymes. We found that roughly a third of these genes was indeed located in three different types of genomic clusters: 17 and 26 genes were each found in small clusters located in CEC and NCEC areas, respectively, and another 27 in MCC. The types of enzymes encoded by the genes in these three types of chromosomal locations did not allow an assignment of a specific function to these three clusters. Yet most of the genes present in CEC and NCEC encoded cellulolytic enzymes: most noteworthy are CEL7A (cellobiohydrolase I), CEL6A (cellobiohydrolase 2) and CEL5B (endo-β-1,4-glucanase EGLB2), which make up for a significant part of the cellulolytic activity of *T. reesei*, but also the accessory protein swollenin, GH3 β-glycosidases and enzymes cleaving the side chains in hemicelluloses and pectin. Also interesting was the absence of xylanase-encoding genes of family GH10 and GH11, whereas the two GH30 xylanases, which are active on substituted xylans [60], were present in CEC. This suggests that a significant part of *T. reesei’*s activity on plant biomass degradation is encoded by CEC/NCEC-located genes. This high number of CAZyme genes contrasts with those present near the subtelomeres in *N. crassa* and *M. oryzae* (six and three genes, respectively; [34, 61]). Wu et al. [34] interpreted the higher number in *N. crassa* compared to *M. oryzae* as the difference between a saprotroph (which derives carbon by degrading the walls of dead and dying plant cells) and facultative biotroph. Yet the difference between saprotrophic *N. crassa* and mycotrophic–saprotrophic *T. reesei* was even stronger (6 vs. 20 genes, respectively). This difference may be explained by the fact that *N. crassa* colonizes burned vegetation, a condition where not too many competing organisms may initially be present, and therefore a still limited reservoir of CAZyme genes may suffice for growth of the fungus. In contrast, *T. reesei* is not only a bio- and necrotrophic mycoparasite but is also a secondary colonizer of dead wood where it likely follows wood-decay fungi that it may also prey on [23]. Under such conditions, an increased arsenal of both degrading (cellulase, hemicellulases) as well as defending (chitinases) enzymes may be beneficial. It is interesting in this regards that the more specialized, albeit likely CAZyme-dependent, nutrition of *N. crassa* may be powered by a smaller arsenal of these enzymes compared to *T. reesei*, which is ecologically more versatile [24].

Despite of the fact that a large portion of the CAZyme encoding genes were organized as clusters, the gene encoding the major transcriptional regulator of their expression—*xyr1* (Trire2:122208)—is not clustered with any of the CAZymes but located in the centre of Chr1 in a gene neighbourhood unrelated to cellulose and hemicellulose utilization. While surprising at a first glance, this may make sense for its function: Klaubauf et al. [62] have shown that the XYR1 orthologues of most fungi (*N. crassa, A. nidulans, M. oryzae, Fusarium graminearum*) only regulate some hemicellulase—particularly xylanase—gene expression. In contrast, *T. reesei* XYR1 also controls the expression of all cellulase and most hemicellulase genes [53, 63]. Under such conditions, an inclusion of *xyr1* in one of the several CAZyme clusters would probably restrict its expression under all required conditions, and thus its independent location can be advantageous.

Although both CEC and NCEC were located within 150 kb of the chromosome ends (representing 2–4 % of their size), our analysis allowed to distinguish between CEC and NCEC. Our main reason for doing so came from the assumption that the “true” subtelomeric genes occupy maximally 50 kb. In addition, the distribution of clusters in CEC and NCEC was not a continuum, but formed two groups separated by about 20 kb between them. Further support for distinguishing between CEC and NCEC was also obtained from the expression analysis of the CAZyme clusters located there and the distribution of histone H3 methylation: the genes located in CEC had a significantly lower basal (non-induced) expression level than those in NCEC, and while CEC was enriched in H3K9 methylations, NCEC was completely devoid of it. Mammalian telomeres and subtelomeric regions are enriched in epigenetic marks that are characteristic of heterochromatin [64], and Smith et al. [65] demonstrated that H3K9 methylation is responsible for telomeric silencing in *N. crassa*. The fact that the CEC located genes are strongly repressed under non-induced expression conditions is therefore likely the result of epigenetic silencing. This finding could have potential biotechnological prospects for strain engineering in *T. reesei*: our data suggest that placing a promoter into any of the CEC areas may lead to a tight shut-off of expression under non-induced conditions. Such expression systems are strongly looked for in industry, but only one has recently been published for *T. reesei* [66].

The genes located immediately after the subtelomeric sequences are believed to be relevant to habitat adaptation of the organism [14, 56, 67], likely because these areas represent major hotspots for recombination [68, 69]. We thus took a closer look into their gene content to identify gene families other than CAZymes that are enriched in the *T. reesei* CEC. Indeed, genes encoding proteolytic enzymes and amino acid transport made up for a major part. *T. reesei* has been shown to utilize proteins and peptides preferentially to cellulose [70], which is in agreement with the original mycoparasitic nature of the genus *Trichoderma* [24]. Feeding on the biomass of other fungi degrading the wood [23] would aid in bypassing the limitations posed by the low nitrogen content of lignocellulose.

In addition, it was striking to detect more than half of all genes related to iron scavenging to be located in CEC. Based on the high expression of some iron scavenging genes by *T. reesei* during growth on cellulose, we have previously speculated that the fungus may involve iron in its mechanism for degradation of cellulose [52]. However, it is also possible that the abundance of iron uptake systems serves *T. reesei* to quickly withdraw iron from its environment, thereby depriving potential competing organisms from it. Such a strategy has been detected in animal and plant pathogenic fungi as well [71], and would not be surprising to occur in an opportunistic fungus like *Trichoderma*.

*Trichoderma reesei* CEC also contained the high affinity nitrate assimilation cluster, which has been acquired by horizontal gene transfer from smut fungi [49]. Slot and Hibbett [49] showed that the nitrate assimilation cluster in the Sordariomycetes first disassembled and was finally lost in *T. reesei*. They speculated that the acquisition of the basidiomycete nitrate assimilation cluster could have provided a benefit once *T. reesei* specialized for growing on decaying wood; as already mentioned above, wood contains only very little nitrogen, but the primary basidiomycete decomposers of wood provide not only protein (vide supra) but also contain an increased nitrate content [72]. Its use as a nitrogen source could have enabled *T. reesei* to enhance its growth rate and thus competitive abilities in this habitat.

Although we did not find major differences in gene expression under cellulase and hemicellulase inducing conditions between CEC, NCEC, MCC and randomly distributed genes, a significantly higher portion of CEC genes was expressed under conditions of confrontation with another fungus, and during the onset of asexual sporulation. Interestingly, also a significant portion of the SSCP clusters was exclusively expressed at the onset of asexual sporulation, and a subset was also expressed during growth on cellulose, but not under any other condition. Expression of SSCPs under conditions of conidiation has so far not been reported for any fungus. Clusters of SSCPs have first been detected in *U. maydis* [5], and subsequently in many other fungi, especially plant pathogenic, plant symbiotic or nematophagous fungi [73, 74]. Functional analysis of selected members showed that they manipulate the cellular processes in the hosts to facilitate infection [75, 76]. It would have therefore been tempting to explain their presence in *T. reesei* as tools assisting in the mycoparasitic attack of other fungi and defence from them. However, our data argue against this interpretation, as none of the SSCP-containing clusters was expressed during confrontation with *T. cucumeris*. The function of SSCPs has not yet been systematically studied in *Trichoderma*, but *T. atroviride* EPL1—a member of the SSCP subclass cerato-platanin—has been shown to exhibit surface modulating and chitin-binding activities [77, 78]. If other SSCPs also exhibit surface binding properties, it may present an advantage to the conidia in attaching to a potential habitat. The fact that several of the SSCP clusters occur in CEC and contain also genes encoding CAZymes and proteolytic enzymes supports the view that these proteins also contribute to the fitness of *T. reesei* in its habitat.

## Conclusions

The availability of fully annotated chromosomal maps of *T. reesei* will facilitate the selection of marker genes for sexual crossings [79], e.g. by identifying several amino acids, nucleotide or coenzyme biosynthesise genes for each of the chromosomes, whose knock out will lead to auxotrophic strains. Correlation of their segregation in the progeny of mating with that of still unresolved phenotypes may help in the identification of responsible genes for the latter. Genes responsible for RNA processing and modification, amino acid metabolism, transcription, translation and ribosomal structure and biogenesis and a third of the genes encoding CAZymes occurred in loose genomic clusters. The latter also contained a high number of genes encoding SSCR proteins. Five CAZyme clusters, including the genes encoding the major cellulases CEL7A, CEL6A, CEL5B, were located less than 50 kb apart from the chromosome ends. The genes present in these putative subtelomeric areas reveal very low basal gene expression, which could be used in design of conditionally operating expression systems. These areas were also enriched in genes encoding secreted proteases, amino acid permeases, enzyme clusters for PKS–NRPS hybrids and proteins involved in iron scavenging. They were strongly upregulated during conidiation and interaction with other fungi, thus stressing their importance to the fitness of *T. reesei* in its habitat.

## Methods

### Complete manual annotation of the *T. reesei* genome

We used a completely manually curated annotation of *T. reesei* QM6a in this work. This was obtained by re-analysis of all not identified or ambiguously annotated genes deposited at the *T. reesei* genome website (http://genome.jgi-psf.org/Trire2/Trire2.home.html). To this end, we used BLASTP against the NCBI database (last accession July 12, 2015), and used only hits with *E* values <e−100 (Hypocreales) or <e−75 (other Pezizomycotina) for identification. In the latter case, i.e. where a protein has been identified by high similarity only to a fungus outside the Hypocreales, a phylogenetic analysis was performed to test whether the protein is indeed an orthologue (D. Yang, C.P. Kubicek, I.S. Druzhinina, manuscript in preparation). In all those cases where the protein has orthologues in other fungi, but its function is as yet unknown, the protein was termed “unknown protein”. Proteins that were only present in *T. reesei* or in other *Trichoderma* spp., but absent from other fungi (using a cut-off of >e−30) were considered “orphan proteins”. For orphans with no ESTs in the JGI and NCBI database, we reinvestigated whether their reading frame was correct. While several cases of incorrect annotation were indeed detected, this did not result in a change from “orphan” to “unknown” or already identified genes (unpublished data). As a last step, we mapped the annotated genes on the seven chromosomes, using the GRAAL-supported assembly of the *T. reesei* scaffolds [22]. The resulted database is available at: (http://trichocode.com/index.php/t-reesei).

### Analysis of genomic clustering of genes

To test whether the genes involved in similar cellular functions would show non-random distribution in the genome of *T. reesei*, we first grouped the *T. reesei* genes according to the KOG classification scheme (http://genome.jgi.doe.gov/cgi-bin/kogBrowser?db=Trire2). This resulted in 5754 genes that were contained in 19 groups. The potential clustering of each of these groups on the seven chromosomes was then tested by a manual sliding window approach. The size of the window was thereby chosen as follows: we divided the total number of ORFs in the *T. reesei* genome (9194) by the number of genes falling into a given above group. This quotient was then taken as the theoretical average distribution of genes of this functional category. We then defined a cluster as the occurrence of at least three genes that were on the average separated from each other by less than a fifth of the above determined average distribution number (e.g. in the above example at least three genes within a stretch of thirty genes). The window of this size was moved stepwise by one gene until the entire chromosome was covered. The statistical significance of the observed non-random occurrence within a window was calculated by the Student’s *t* test (http://studentsttest.com/), assuming unequal variance of groups [80].

Tests for random or non-random occurrence of CAZymes was done in the same way, using a window size of eight (average random distribution of CAZymes is one in 41).

To identify clusters of secreted proteins of *T. reesei,* we used the stringent approach introduced by Kämper et al. [5]: groups of at least three adjacent genes encoding secreted proteins or groups containing more than three genes with at most one gene encoding a non-secreted protein in between.

### Transcriptome analysis

We used transcriptome data from our own earlier work. These included: cultivation of *T. reesei* QM 9414, an early cellulase producing mutant, on d-glucose, glycerol, lactose and wheat straw (mechanically ground, and subjected to slightly acidic, thermochemical pre-treatment; obtained from Clariant Produkte Deutschland GmbH), respectively, in batch cultures [51–53], during induction of conidiation [58], induction of cellulase gene expression by sophorose [53] and at the onset of confrontation with the basidiomycete *Thanatephorus solani* [57]. All transcriptome data were obtained by oligonucleotide array hybridization, with the exception of the data for cultivation on glycerol and induction by sophorose, which were obtained by RNA deep sequencing. For the former, a high-density oligonucleotide microarray (Roche-NimbleGen, Inc., Madison, WI) with 60-mer probes representing 9129 genes of *T. reesei* was used. Values were normalized by quantile normalization [81] and the RMA algorithm [82]. After elimination of transcripts that exhibited an SD >20 % of the mean value within replicates, false discovery rates ([83] were used to assess the significance of values. Data from RNA deep sequencing were analysed using the EOULSAN software version 1.2.2 [84]. To quantify the gene expression level, the relative transcript abundance was measured in reads per kb of exon per million mapped sequence reads (RPKM; [85]). All transcriptome data and the related protocols are available at the GEO web site (http://www.ncbi.nlm.nih.gov/geo/) under the accession numbers given in Table 7.

**Table 7:** Accession numbers for transcriptome data used in this paper

Condition	Accession number	Method	Ref.
Glucose vs. lactose	GSE39276	Oligonucleotide array	[51]
Glucose vs. cellulose (wheat straw)	GSE46155	Oligonucleotide array	[52]
Glycerol, sophorose	GSE59600	RNAseq	[53]
Conidiation	GSE27471	Oligonucleotide array	[58]
Confrontation	GSE23438	Oligonucleotide array	[57]
Lactose for ChiP-sequencing	GSE22687	Oligonucleotide array	[54]

### Chromatin immunoprecipitation (ChIP) and ChIP-sequencing

Chromatin immunoprecipitation (ChIP) and ChIP-sequencing data were taken from our earlier work, using cultures growing on lactose [54]. The antibodies used were from Active Motif (H3K4me3, 39159; H3K9me3, 39161) and Millipore (H3K4me2, 07-030).

### Bioinformatic analyses

Repetitive and transposable elements were detected using CENSOR [86]. Clusters for synthesis of secondary metabolites were identified by antiSMASH [46]. The GC content of the chromosome ends was calculated and plotted by the *Z*-curve analysis, a window-less approach [36].

### Statistic tests

The significance of differences in numbers of clusters or the gene expression level between MCC, NCEC, CEC and “randomly distributed genes” was evaluated by the Student’s *t* test (http://studentsttest.com/), assuming unequal variance of groups. To this end, a group consisting of the non-randomly distributed genes or transcripts of a given family was compared against a group containing all genes or transcripts of this family in the genome.

### Data availability

All microarray files are available from the National Center for Biotechnology Institute Gene Expression Omnibus (GEO) repository database, as given in Table 7. The complete chromosome annotation is available on http://www.e166.org/tools/trichocode/reesei/index.php and on http://trichocode.com/index.php/t-reesei. All other relevant data are within the paper and its supporting information files.

## Additional files

10.1186/s13068-016-0488-z *Z*-curve analysis of AT rich regions in CEC. ND, no segmentation point detected.
10.1186/s13068-016-0488-z CAZyme clusters on *T. reesei* chromosome. * NG: not given for genes located at >200 kb from either chromosomal end.
10.1186/s13068-016-0488-z SSCP clusters in *T. reesei.* * CAZymes are indicated in italics; SSCPs in bold.
10.1186/s13068-016-0488-z Putative structure of the two PKS–NRPS products as predicted by antiSMASH [47] (A) and genomic organization of the two telomeric PKS–NRPS clusters (B). Abbreviations used: AT: Acyltransferase; KS: Keto-synthase; KR: Ketoreductase; DH: Dehydratase; cMT: carbon methyltransferase; C, condensation domain; A, activation domain; TD, thioesterase domain. The blue boxed domains do not specify any enzymatic function. R1–R3 in the chemical formulae specify a not-predictable substituent. Genes are indicated by Trire2: IDs, and shown in 5′→ 3′ order.
10.1186/s13068-016-0488-z Transposable elements in CEC regions of *T. reesei* chromosomes.

## Abbreviations

CAZymes: carbohydrate-active enzymes
PKS: polyketide synthase
NRPS: non-ribosomal peptide synthase
SSCP: small secreted cysteine-rich protein
MCC: main chromosome located clusters
NCEC: near chromosome ends clusters
CEC: close to chromosome ends clusters
GH: glycosyl hydrolase
