# Supplementary figures and images for: A complete annotation of the chromosomes of the cellulase producer Trichoderma reesei provides insights in gene clusters, their expression and reveals genes required for fitness
Source: Biotechnol Biofuels. 2016 Mar 29;9:75. doi: 10.1186/s13068-016-0488-z (PMC4812632; doi:10.1186/s13068-016-0488-z)

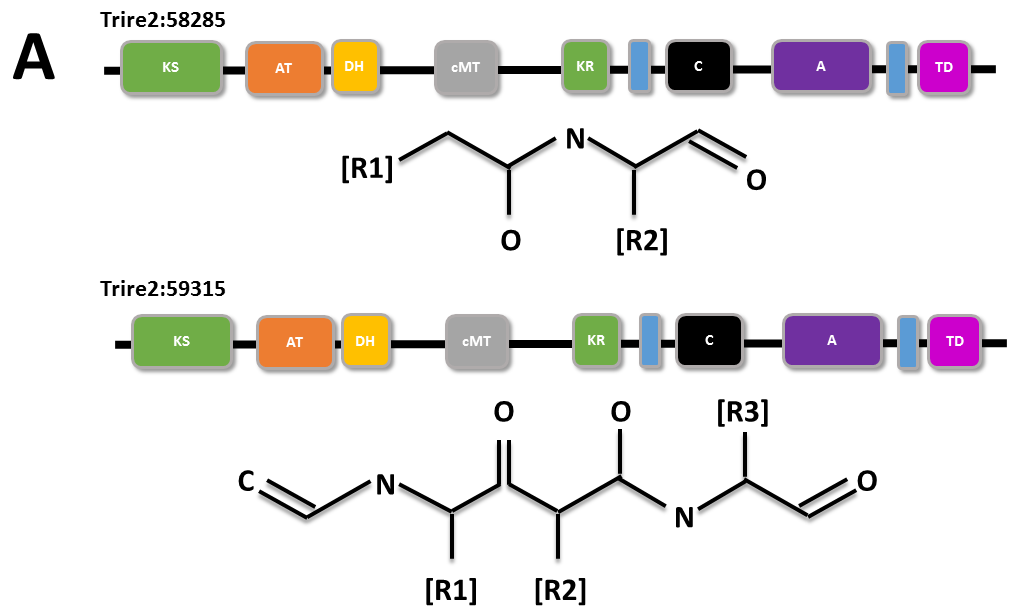


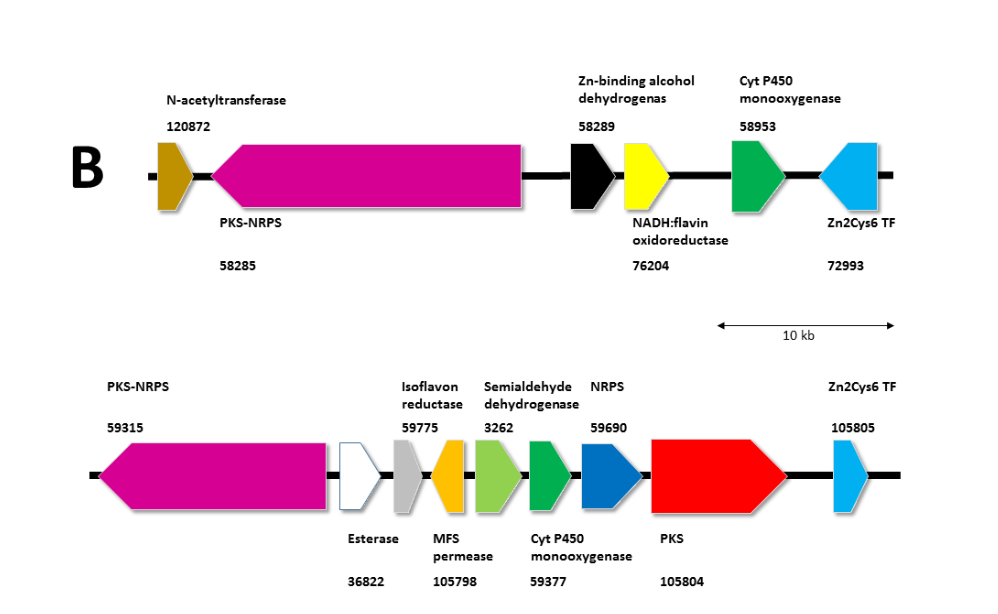

Supplement: Supplementary file 1 — 10.1186/s13068-016-0488-z Z-curve analysis of AT rich regions in CEC. ND, no segmentation point detected. [file 13068_2016_488_MOESM1_ESM.doc]
